# Toxicokinetic study following intratracheal instillation or oral gavage of two [^7^Be]-tagged carbon black samples

**DOI:** 10.1186/s12989-022-00504-8

**Published:** 2022-10-14

**Authors:** Otto Creutzenberg, Volker Hammann, Stefanie Wolf, Jürgen Daul, Yufanyi Ngiewih, Ishrat Chaudhuri, Len Levy

**Affiliations:** 1 Fraunhofer Institute for Toxicology and Experimental Medicine, Hannover, Germany; 2 Zyklotron AG, Karlsruhe, Germany; 3 Orion Engineered Carbons GmbH, Cologne, Germany; 4 Cabot Corporation, Boston, MA USA; 5 Cranfield University, Cranfield, UK

**Keywords:** Carbon black, Intratracheal instillation, Oral gavage, ^7^Be tracer, Toxicokinetics

## Abstract

**Background:**

The toxicokinetic behaviour of nanostructured particles following pulmonary or oral deposition is of great scientific interest. In this toxicokinetic study, following the general principles of OECD TG 417, the systemic availability of carbon black, a nanostructured material consisting of agglomerated aggregates was characterised.

**Methods:**

Each of two grades of beryllium-7 labelled carbon black (Monarch® 1000, oxidized and Printex® 90; untreated) was administered either intratracheally or orally to adult rats. Independent of route, rats received a single dose of approximately 0.3 mg radiolabelled carbon black. A total of 12 rats were treated per grade and per exposure route: 4 females each for feces/urine/organs and serial blood kinetics; 4 males for organs. At necropsy, the complete suite of organs was analysed for females, but only the lungs, liver, kidney, reproductive organs for males.

**Results:**

In the pulmonarily exposed animals, ^7^Be-Monarch® 1000 and ^7^Be-Printex® 90 was detected in feces in the first 3 days after treatment at significant levels, i.e. 17.6% and 8.2%, respectively. In urine, small percentages of 6.7% and 0.4% were observed, respectively. In blood, radioactivity, representative of carbon black was within the background noise of the measurement method. At necropsy, 20 days post-instillation, both test items were practically exclusively found in lungs (75.1% and 91.0%, respectively) and in very small amounts (approximately 0.5%) in the lung-associated lymph nodes (LALN). In the other organs/tissues the test item was not detectable. BAL analyses indicated that carbon black particles were completely engulfed by alveolar macrophages.

In orally exposed animals, 98% (^7^Be-Monarch® 1000) and 99% (^7^Be-Printex® 90) of the measured radioactivity was detected in feces. Excretion was complete within the first 3 days following treatment. 1.3% and 0.5% of measured activity was attributable to urine in animals that received ^7^Be-Monarch® 1000 and ^7^Be-Printex® 90, respectively. Radioactivity was absent in blood and other organs and tissues.

**Conclusion:**

Radioactivity, representative of carbon black, was not detected beyond the experimentally defined limit of quantitation systemically after deposition in lungs or stomach in rats. Under these experimental conditions, the two CB samples were not shown to translocate beyond the lung or the GI tract into the blood compartment.

**Supplementary Information:**

The online version contains supplementary material available at 10.1186/s12989-022-00504-8.

## Background

Carbon black [CAS. No. 1333–86-4] is elemental carbon in the form of particles that are produced industrially by the partial combustion or thermal decomposition of gaseous or liquid

hydrocarbons under controlled conditions. The physical appearance of carbon black is that of a black, finely divided powder, consisting of aggregates (in the size range between 100 and 1000 nm) of aciniform morphology (i.e. aggregates that have been strongly fused together in random configuration that resemble grape-like clusters) [1]. Constituent particles (ranging from 10 to 500 nm), which are defined according to ISO as “the original source particles of aggregates or agglomerates or mixtures of the two” are not discernible anymore after completion of the manufacturing process. The aciniform aggregates constitute the smallest inseparable entities in manufactured carbon black and are hence the fundamental structural units of carbon black. However, even the carbon black aggregates are not readily available outside the closed reaction chamber of the manufacturing process as, within the reaction chamber, the aggregates rapidly form larger agglomerates held together by van der Waals forces. A large fraction of carbon black is used in the rubber industry, mainly in the manufacture of tires. A small fraction is used in non-rubber applications such as paints, inks, coatings, plastics, electrostatic discharge compounds, ultraviolet light absorption applications, and as a chemical reagent. Agglomerate sizes measured after dispersion in aqueous media (e.g. in artificial lung fluids) usually occur in the microscaled range.

The toxicity of carbon black has been widely investigated; often carbon black was used as a reference material for carbonaceous particles, e.g. in the research on diesel engine exhaust. However, it is important to differentiate carbon black from “soot” or “black carbon” which are names applied to carbonaceous emissions from fires and incomplete combustion of carbon-containing fuels and materials. Such emissions are not only comprised of some elemental carbon but also of significantly higher quantities of organics and other compounds [2]. The main health concerns associated with carbon black and other poorly soluble, low-toxicity (PSLT) particles are lung effects resulting from inhalation exposure. In the rat lung, studies have shown that particle deposition above certain concentrations lead to a phenomenon known as “lung overload”, which in turn leads to sustained inflammation, production of reactive oxygen species (ROS), depletion of antioxidants and/or impairment of other defense mechanisms, and eventually lung tumors in the rat [3]. A number of recent studies have suggested that carbon black may have hazardous effects that fall outside the well-understood mechanism of action for PSLT particles; for example, that carbon black may be directly genotoxic in organs that lie beyond the site of contact, and that it may cause reproductive toxicity (reviewed in [4]). These adverse effects presuppose that carbon black particles can translocate into the blood stream and to travel from the original point of contact to distant target organs. For spark generated carbon aerosols labelled *in statu nascendi* with Iridium-192 (so-called composite iridium-labelled carbon NP with an aggregate particle size of approx. 25 nm) a translocation to secondary target organs was detected amounting to approx. 0.5% of the deposited dose of approx. 1 µg in lungs [5]. The purpose of this toxicokinetic study was to determine whether carbon black may be absorbed or distributed systemically in the body and if so, to what extent. Both pulmonary and oral uptake were evaluated, as these are the two most relevant pathways of exposure.

While some authors have reported the use of optical imaging methods to identify carbon black in in vivo systems [6], reliable tracking and quantification of carbon black in biological systems remains technically challenging as a chemical analysis of carbon would not allow for a sharp differentiation between carbon black-derived carbon and the carbonaceous background of organic matrices. To overcome this hurdle, we applied the methodology, used and described by Lefevre et al. [7] who successfully applied radiolabelling of the γ-tracer, Beryllium-7, to study the toxicokinetic behaviour of carbon black. Small scale production of beryllium 7-labelled carbon black has been described by Abbas et al. [8] and Bäcker et al. [9]. We applied these methodologies, having made appropriate modifications, to generate the radiolabelled material used in our studies. Successful accomplishment of the study goal is tethered to the stability of the bond between carbon black and the chosen label. Notably, leakage of the label into physiological fluids is not permissible as this would compromise the interpretation of study results. The stability of the bond between carbon black and beryllium-7, which is embedded within the amorphous carbon matrix of carbon black, was ascertained before use of the test item in animal experiments. We applied a study design that followed the general principles set out in OECD 417 for toxicokinetic studies.

### Objectives of the study

The toxicokinetic fate of two commercial carbon black grades on the inhalation and oral pathway was investigated. The objectives of the study were.

- To generate toxicokinetic data to answer questions about potential systemic translocation and to help underpin and understand any possible mode(s) of action for systemic toxicity;
- To compare two high-volume grades of carbon black of similar constituent particle sizes, one without (Printex® 90) and the other with surface functionalization (oxidized) (Monarch® 1000);
- To assess any translocation, distribution and excretion of carbon black beyond the target organ, i.e. lung and gastro-intestinal tract.

## Methods

### Test materials

Test item information is given in Table 1. To elucidate the possible effects of oxidative treatment of carbon black on toxicokinetic behaviour, two grades, one treated and one untreated, but with similar particles sizes and surface areas were selected for the study. The carbon black samples Monarch® 1000 and Printex® 90 were provided by Cabot Corp., Billerica MA, U.S.A. and Orion Engineered Carbons GmbH, Frankfurt, Germany, respectively.

**Table 1:** Test item data and properties

Name Trade name Purity Specific surface (m^2^/g) – BET CAS number Surface functionalization Average constituent particle size Manufacturer	^7^Be-tagged monarch® 1000 Carbon black, amorphous Monarch® 1000 100% 340 1333–86-4 Yes; oxidized 16 nm Cabot corporation	^7^Be-tagged printex® 90 Carbon black, amorphous Printex® 90 100% 350 1333–86-4 No 14 nm Orion engineered carbons

### Particle agglomerate sizes in suspension media

The purpose of this initial test was to determine the size of the particles in biological media, including artificial alveolar fluid (AAF) and artificial lysosomal fluid (ALF), and to compare the non-labelled and ^7^Be-labelled material. The particle agglomeration size determination was conducted using a Sympatec SUCELL device (Sympatec GmbH, Clausthal-Zellerfeld, Germany). The laser diffraction sensor HELOS/KR covers a wide range of particle sizes with its long optical bench (from 0.1 to 1,750 µm). The carbon black samples were suspended in 15 ml polypropylene tubes amounting to concentrations of 1 mg/ml. This value corresponded to the concentration used for instillation dosing of rat lungs in this toxicokinetic study. A 1-min ultrasonic treatment was followed by vortexing (10 s). For an optimal measurement in the SUCELL cuvette, these stock suspensions had to be diluted with the respective medium in the ratio 1:10 v/v. Following dilution only repeated short vortexing and manual shaking but no sonication was applied to maintain the agglomeration status of the preceding preparation step. In the case of AAF, this medium was too cloudy for measurement; thus the dilution step 1:10 v/v was done with saline. 10 ml each of the agglomerate suspensions were filled into the cuvette and 3 runs of the particle size analysis were performed using a Sympatec HELOS—Laser Diffraction Spectrometer.

Using much lower concentrations to allow dynamic light scattering (DLS) analysis (ZetaSizer, Malvern, Worcestershire, Great Britain), hydrodynamic diameters of a Monarch® 1000 suspension in 0.01 N NaOH were determined and compared to ^7^Be-Monarch® 1000.

### Production and purification of radioactively labelled carbon black

In order to monitor the passage of carbon black systemically, it was necessary to use a radiolabel as carbon black’s identification and quantification against a carbonaceous background would otherwise not be possible. Radiolabelling of carbon black can be achieved by using radioactive precursor substances for the synthesis or by direct irradiation of carbon black. In this study, the method of direct irradiation described was applied [7–9]. These studies have shown that carbon black can be intrinsically labelled with a *γ*-tracer without evidently altering the material structure. Through proton irradiation, the ^7^Be radioisotope is produced (nuclear reaction: natC(p,x)^7^Be, mainly via ^12^C(p,3p3n)^7^Be channels). The γ tracer ^7^Be with a half-time of 53 days is a very useful radioisotope in biokinetic studies.

The optimal conditions on beam current and target cooling were provided kindly by Uwe Holzwarth, JRC, Ispra, Italy.

- Approximately 15–20 mg carbon black was filled into a target aluminium capsule and the cavity sealed with a screwed cap.
- Target specific activity: 150 kBq ^7^Be/mg
- Proton energy: approx. 30.9 MeV
- Beam current of 10 µA (approximately 10 h beam time)

Proton irradiation of carbon black was performed at ZAG Zyklotron in Karlsruhe, Germany.

Prior to dosing, it was important to ascertain that no leakage of the tracer (i.e., ^7^Be) from the carbon black particles is occurring as this would compromise the interpretation of the toxicokinetic behaviour. Therefore, a purification step was performed before using the test item in animal experiments. To remove soluble or loosely attached moieties of the radiolabel from the surface of the carbon black test item, a triplicate application of solvents (EtOH/H_2_O 1/1 v/v – 0.01 N HCl – Artificial lysosomal fluid—ALF) was performed consecutively to wash and filter the test item [7, 8]. An aqueous suspension of approx. 0.3 mg of the ^7^Be-carbon black sample in a syringe was suspended in water and the carbon black separated by pressing the suspension smoothly through a filter with the thumb (pore size: 0.4 µm; Swinnex® Filtration System CAT # SX0001300, EMD Millipore, U.S.A.). A volume of 10 ml of each of the three washing media was used for purification. The completeness of the purification step was confirmed by measuring the eluates gained in aliquots after filtration for radioactivity.

### Test system

Female Wistar rats [strain: Crl:WI (Han)] were used for the comprehensive animal tests (feces/urine collection, blood kinetics, organs/tissues). In addition, one subset of males of the same strain was also included. The rats were acclimatized for 2 weeks in the animal house and were approx. 9 weeks of age at treatment. Individual body weights were recorded to the nearest 0.1 g twice a week throughout the study for all animals. All body weight data were collected using electronic balances and documented in datasheets. The animal studies were approved by the competent authority (file # 33.9–42,502-04–18/3019/LAVES, Oldenburg, Lower Saxony).

### Study design and dosing

The general principles of OECD TG 417 were followed for the design and performance of the study. For pulmonary exposure, intratracheal instillation was chosen over inhalation because only small absolute amounts of radiolabelled carbon black could be generated; approximately 20 mg of carbon black could be activated per run and the short half-life of the radiolabel precluded accumulation to achieve larger quantities. Prior to use, intense washing of the radiolabelled product was performed such that leaching, which would hamper interpretation of the results could be excluded.

### Intratracheal instillation and dosage of the test items

The rats were anaesthetised with isoflurane and treated by a single intratracheal instillation of approximately 0.3–0.4 mg carbon black (depending on the specific radioactivity) suspended in 0.3 ml of sterile isotonic saline. This lung load for carbon black was calculated to be below the “lung overload levels” so as not to compromise the physiological ADME capability [10–13].

Instillation was preferred to inhalation as only limited amounts of radiolabelled test items could be generated, and for safety reasons, only small amounts of radioactive material could be handled.

### Oral administration and dosage of the test items

The test item was dispersed in tap water (approx. 0.3 mg carbon black in 1.5 ml tap water) and administered once by oral gavage to the rats. Minimal but visible losses of ^7^Be-carbon black in the plastic ware occurred, however, the highest loss of the nominal dose is considered to be due to a particle “cake formation” in the syringe. A cannula was used for administration. As the radiolabelled test item was available at limited amounts only, the same dose was used for oral administration as was used for intratracheal administration.

### Collection of feces and urine

Following administration, rats were held in metabolic cages capable to collect feces and urine separately. For reasons of animal welfare, the daily housing in these cages was limited to 19 h/day.

### Blood kinetics

Radiolabelled test items were administered to the rats and blood samples were collected serially from the tail vein for up to day 4. The time-points chosen were 2, 5, 8, 24, 30, 48 and 72 h after administration. At final sacrifice (e.g. day 20), total blood was collected as well.

### Sacrifice of rats

Rats scheduled for toxicokinetic analysis were sacrificed by cutting the *vena cava caudalis* after anaesthesia with an overdose of pentobarbital sodium (Narcoren®).

### Collection of organs for radioactivity analysis

Following necropsy on day 20, the following organs of female animals (Table 2) were prepared and preserved until analyses. A limited number of organs (testes, epididymides; stomach, intestine, liver, kidneys, blood) were also prepared and preserved from the satellite group of male rats.

**Table 2:** Organs under analysis in females and males

Females	Males
Lungs, lung-associated lymph nodes, liver, kidneys, adrenals, fat, brain, eyes, stomach, intestine, colon, heart, muscles, brain, spleen, femur, bone marrow, thyroids, whole blood—ovaries, uterus—residual carcass (3 locations: skin, ear, tongue)	Lungs, liver, kidneys, whole blood—epididymides, testes –stomach, intestine

### Measurement of radioactivity

^7^Be *γ*-activity of blood, organ, tissue and excreta samples were analysed using an automatic *γ* counter (Perkin Elmer 1470 WIZARD) including always a repeat determination of counts per minute (double counting). The general background A of the gamma counter was 33.5 cpm. This value A is automatically subtracted from the measured values by the gamma counter. In serial countings, the concurrent blind vials without test item-related radioactivity resulted in mean deviation values of 0.8 ± 1.6 cpm (Intratracheal (IT) Monarch 1000, *N* = 28), 1.1 ± 1.5 cpm (IT Printex 90, *N* = 24), 1.4 ± 3.2 cpm (Oral gavage(OG) Monarch 1000, *N* = 20), 0.6 ± 1.4 cpm (OG Printex 90, *N* = 19) background B. The given values for tissues, urine/feces in the tables S1-S8 are not background B-corrected; cpm-values in the range 2.4–4.6 cpm are considered to be below the limit of quantitation (LOQ), i.e. not test item-related. Thus, the probability of statistically confirmed translocation of nanoscaled particles is minimal.

Directly before intratracheal instillation or oral gavage, the radioactivity of the test item suspension in 300 µl saline or 1500 µl water was measured. For mass balance evaluations, it is important to ascertain the true dose administered to the test animals. Therefore, mock administrations were performed to determine how much test material (calculated nominal concentration) may be lost to the system during the administration process (e.g., residual material in syringes and other vessels). The results of these tests indicated that average losses of the nominal dose could be as high as 40%. Unlike fully soluble substances, insoluble substances such as carbon black are prone to form large agglomerates. Further, in syringes, there is a “caking” tendency. Consequently, depending on the actual flow behaviour of the carbon black suspension in the syringe, variations in expulsion are inevitable. Because of the difference between nominally calculated and actually administered concentrations, the presented results of this study are not calculated as percentages of the nomimal dose administered to the rats, but as a fraction of the total radioactivity detected in all rat body compartments combined; this was set as the 100% value.

### Bronchoalveolar lavage (BAL)

Lungs of male rats exposed to Printex® 90 were lavaged and cytospot slides were prepared (spot checks only) to clarify the localization of carbon black after uptake in lungs. This exhaustive BAL (including a gentle massage) was performed in 2 males on day 20 post-treatment. The method of Henderson et al. [14] was used with minor modifications. BAL fluid was centrifuged post-lavage to precipitate the cells; radioactivity in the supernatant and cell sediment were analysed.

## Results

### Purification of test items

The sequential washing with different solvents resulted in a stable radiolabel of the test items. Washing was performed until radioactivity in the filtrate was not detectable (approx. 20 cpm/mL filtrate or 0.1% of the nominal dose). In the following study modules (feces/urine, blood, organs), background leaching of soluble or loosely attached ^7^Be was not detected in any peripheral body compartment, i.e. values were below the limit of quantitation (LOQ) < 2.4–4.6 cpm (see also section “Measurement of Radioactivity”).

### Particle agglomerate sizes in suspension media

Analysis of the size of carbon black agglomerates in various suspension media by laser diffraction spectroscopy showed mean agglomerate sizes in the micrometer range. For Monarch® 1000 the agglomerate sizes were 12.1 µm in saline; 9.2 µm in AAF; 8.7 µm in ALF; and for Printex® 90, the agglomerate sizes were 15.8 µm in saline; 18.5 µm in AAF; 8.8 µm in ALF. These results mirror the agglomeration status of carbon black in the suspensions before they are introduced into the rats. In contact with bodily fluids, the agglomerates may remain or even form large agglomerates, however at this stage, a definitive statement is not possible as the agglomeration and de-agglomeration is a dynamic process.

**Fig. 1:**
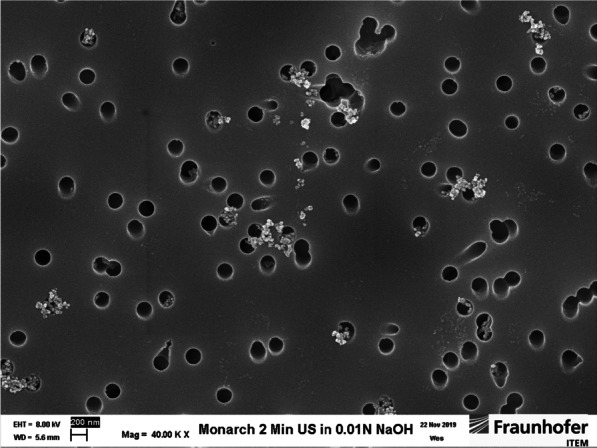
Preparation of Monarch® 1000 suspension in 0.01 NaOH–2 min ultrasonic treatment

Note: Laser diffraction analysis can determine the particle size at a broad size spectrum (nm-µm). DLS analysis can only detect particles in an aqueous suspension that show a permanent Brownian movement; a statistical evaluation is used to calculate mean sizes.

In a separate experiment using dynamic light scattering (DLS) analysis, hydrodynamic diameters of a Monarch® 1000 suspension in 0.01 N NaOH were determined and compared to Be-7 Monarch® 1000 after completion of irradiation. Results revealed that the irradiation process enlarged the mean particle size in suspension (100–200 nm vs. approximately 700 nm), probably because of a compaction effect in the irradiation capsule. However, SEM photographs of the pristine carbon black showed the typical carbon black agglomerates (Fig. 1; Monarch 1000).

### Survival of animals, body weights and gross pathology findings

Unscheduled deaths did not occur during the study. Clinical observations did not show

any signs of toxicity in the animals. Body weights were in the expected range (laboratory historical data) for sex and age. The average values for females were 190–220 g at study start and 210–235 g at study termination; for males, the corresponding values were 280–350 g and 300–400 g, respectively. Necropsy evaluations revealed no significant findings.

### ^7^Be-carbon black levels post intratracheal instillation

#### Feces/urine

The percentage of ^7^Be-Monarch® 1000 in feces and urine on days 1–3 post-instillation reached group mean values (*N* = 4) of 17.6% and 6.7%, respectively (Table 3). For ^7^Be-Printex® 90 the corresponding data were lower and reached 8.2% and 0.4%. On day 4 and thereafter, the measured radioactivity was below the limit of quantitation (LOQ) < 2.4–4.6 cpm (see also section “Measurement of Radioactivity”).

**Table 3:** Radioactivity analysis in feces and urine

Collection on days 1–3 post-treatment	Activity (%) calculated		Activity (%) calculated	
	^7^Be-Monarch® 1000		^7^Be-Printex® 90	
*N* = 4	Mean	SD	Mean	SD
Feces	17.6	18.3	8.2	0.4
Urine	6.7	1.8	0.4	0.3

Percentages refer to the entire radioactivity detected in the rat body (= 100%)

Note: Metabolic cages, when used effectively, separate urine and feces of the rodent into collection tubes outside of the cage. However, there is no guarantee of absolutely uncontaminated samples (from feces to urine by washing over). Radioactivity detected in urine may thus have been from contact with feces rather than via the kidneys through the blood compartment. This conclusion is supported by the lack of radioactivity measured in the blood compartment.

#### Blood kinetics

The two ^7^Be-carbon black grades were not detected at significant levels in blood samples collected on days 1–3. Consequently the derivation of a maximum concentration (c_max_) of the test items in blood (where the organs could have been determined most precisely) is not relevant in this study.

#### Organs/tissues

As the test items were presumably retained in lungs (after intratracheal instillation) or had left the body within few days (after oral gavage), the animals were sacrificed at day 20 post-treatment for analyses of the organs (Table 4).

**Table 4:** Radioactivity analysis in lungs and LALN

Day 20 post intratracheal instillation	Activity (%) calculated		Activity (%) calculated	
	^7^Be-Monarch® 1000		^7^Be-Printex® 90	
*N* = 4	Mean	SD	Mean	SD
Lungs	75.1	23.3	91.0	34.9
LALN	0.6	0.6	0.4	0.3

Percentages refer to the entire radioactivity detected in the rat body (= 100%)

In the intratracheal instillation study, ^7^Be-Monarch® 1000 was detected only in lungs as the deposition site (75.1%; Fig. 3: together with feces and urine amounting to 100%) and, at very low levels (0.6%), in the lung-associated lymph nodes (LALN). Similarly, ^7^Be-Printex® 90 values were 91.0% (Fig. 4) and 0.4% in the lungs and LALN, respectively. In all other organs (including male reproductive organs), measured radioactivity was below the limit of quantitation (LOQ) < 2.4–4.6 cpm (see also section “Measurement of Radioactivity”). (Additional file 1: Tables 1/2 and 3/4).

### Radioactivity measurements in BAL fluid—^7^Be-Printex® 90 levels post intratracheal instillation

**Fig. 2:**
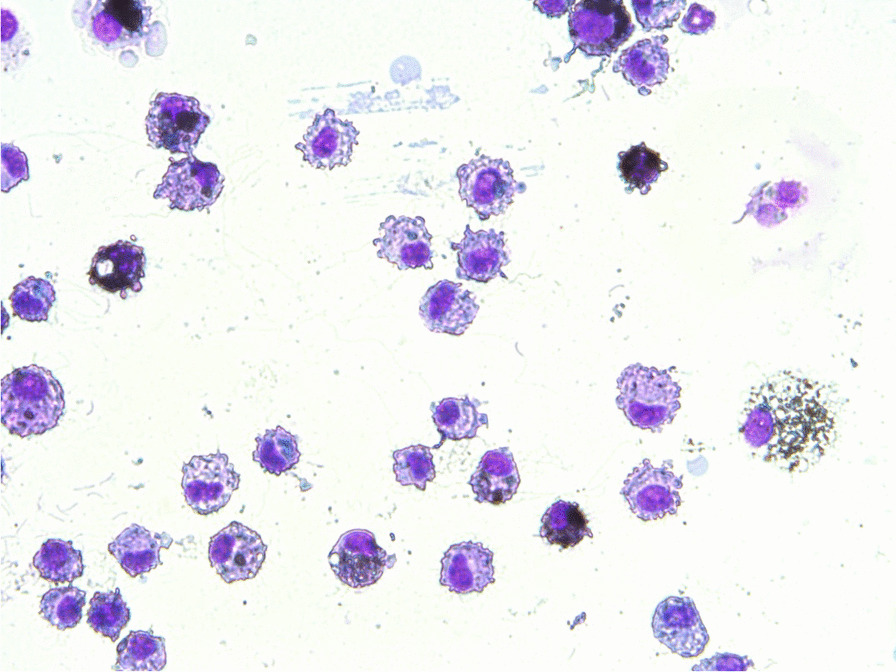
Cytospot of BAL macrophages harvested at day 20 post-instillation (^7^Be-Printex.® 90)

Exhaustive lung lavages were performed to harvest a large moiety of the lung leukocyte pool. Subsequently, the radioactivity of the leukocyte suspension and the remaining lung was counted. These measurements revealed that approximately 50% of the ^7^Be-Printex® 90 originally deposited in the lung had been recovered by the lavage process. Following centrifugation of the bronchoalveolar lavagate, 100% of the ^7^Be-Printex® 90 was localised in the cell sediment, and 0% in the cell-free supernatant. Photographs of cytoslides with lung leukocytes showed that the carbon black was fully engulfed by alveolar macrophages (Fig. 2). The separation of supernatant and leukocytes of bronchoalveolar lavage fluid (BALF) following sacrifice of rats revealed that the radioactive tag was tightly embedded in the turbostratic structure of the carbon black samples.

## ^7^Be-carbon black levels post oral gavage

### Feces/urine

The percentage of ^7^Be-Monarch® 1000 in feces and urine on days 1–3 post-instillation reached group mean values of 97.6% and 1.3%, respectively (Table 5). For ^7^Be-Printex® 90 the corresponding data were similar and reached 99.2% and 0.5%, respectively. On day 4 and thereafter, measured radioactivity was below the limit of quantitation (LOQ) < 2.4–4.6 cpm (see also section “Measurement of Radioactivity”).

**Table 5:** Radioactivity analysis in feces and urine

Collection on days 1–3 post-treatment	Activity (%) Calculated		Activity (%) Calculated	
	^7^Be-Monarch® 1000		^7^Be-Printex® 90	
*N* = 4	Mean	SD	Mean	SD
Feces	97.6	42.7	99.2	22.0
Urine	1.3	0.9	0.5	0.1

Percentages refer to the entire radioactivity detected in the rat body (= 100%)

### Blood kinetics

The two ^7^Be-carbon black grades were not detected at significant levels in blood samples collected on days 1–3. The measured radioactivity was below the limit of quantitation (LOQ) < 2.4–4.6 cpm (see also section “Measurement of Radioactivity”). Consequently a maximum concentration (c_max_) of the test items in blood (where the organs could have been determined most precisely) could not be identified.

### Organs/tissues

An evaluation of the excretion data showed that most of the administered radioactivity was already excreted in feces and urine within the first 3 days post-treatment. Therefore, animals were sacrificed on day 13 (^7^Be-Monarch® 1000) and day 10 (^7^Be-Printex® 90) post gavage for analysis of the organs (Table 6). At necropsy, only 0.2% ^7^Be-Monarch® 1000 remained in the GIT and, at very low levels (total of 0.9%) in the other organs combined; each secondary organ, individually, did not reach a value that definitely was higher than the LOQ.

**Table 6:** Radioactivity analysis in GI tract and other organs

Day 13/10 post gavage	Activity (%) calculated		Activity (%) calculated	
	^7^Be-Monarch® 1000		^7^Be-Printex® 90	
*N* = 4	Mean	SD	Mean	SD
GI tract	0.2	0.2	0.2	0.1
Other organs (combined)	0.9	0.4	0.2	0.2

Percentages refer to the entire radioactivity detected in the rat body (= 100%)

For ^7^Be-Printex® 90, similar values, i.e. 0.2% and 0.2%, respectively, were detected (Additional file 1: Tables 5/ and 7/8). Similarly, for both test items, no radioactivity was detected in the organs evaluated in males, including reproductive organs.

## Discussion

To facilitate the interpretation of the results of this toxicokinetic (TK) study, we sought to understand whether fundamentally, carbon black agglomerates, the form in which carbon black predominantly occurs when dispersed as airborne particulates, are likely to breakdown into smaller aggregates upon deposition, and when resident in the (deep) lung. Underpinning this line of thinking is the fact that agglomerates are held together by weak van der Waals forces, and theoretically, could disintegrate into smaller particles (agglomerates or aggregates) with a higher propensity for uptake and potential translocation. But, since under normal physiologically-relevant conditions, aggregates will not break down further to release constituent particles, it implies that ultimately, the aggregate size is the relevant size that will determine penetration potential through biological barriers and consequentially, any potential biological response. The two carbon black samples in this TK study consist of aggregates in the size range between 100 and 1000 nm. To simulate the interaction of the particles within the lung with lung fluids, we subjected the non-irradiated carbon black samples to particle size measurement analyses after suspension in the lung fluid simulants, AAF and ALF. Measurements in saline were also performed for comparison. Our experiments showed that the carbon black aggregates, in lung fluid simulants as well as in saline, formed larger agglomerates in the micron size range, e.g. of 10 µm, with the actual size varying due to the dynamic character of the process.

While we recognise that the experimental set-up in no way reflects the complex and dynamic nature of the processes within lungs, the aggregate size of the finished carbon black samples and the results on particle size measurements in lung fluids provide a plausible explanation for non-detectability of a systemic circulation that we observed in the animal experiments. We posit thus that, the particles, upon arrival in the lung, are subject to a dynamic process that engenders agglomerated aggregates, that are too large as to be passable into the blood stream. This proposition is supported by work previously performed at Fraunhofer in which we have shown that stable agglomerates of Printex® 90 are formed in the lungs of rats over a period of 24 h (measured 15 min, 1 h, 4 h and 24 h) after intratracheal instillation [15]. Kreyling’s study [5] used the spark generation approach to obtain airborne particle aggregates with a mean size of approx. 25 nm. At aerosol concentrations of approx.1 µg/m^3^ the tendency of those aggregates to agglomerate was reduced evidently. This approach marks the principal difference to our risk-oriented study design, planned to characterise the “real life” and practical risks associated with the use of industrially-produced carbon black grades with aggregate sizes of 100–1000 nm that have a stronger tendency to build large agglomerates.

Using a sensitive method to follow the passage of carbon black in rats, no evidence (i.e. detection of radioactivity above the detection limit of Be-7) was found for the translocation of the test items beyond the lung or the GI tract into the blood circulation or other body compartments. Given the LOQ of the measurement, it is theoretically possible that trace levels of translocated carbon black may have not been detected under the conditions of this study (estimated maximum value of not-detectable carbon black < 0.1% of nominal dose). In the instillation study, ^7^Be-Monarch® 1000 and ^7^Be-Printex® 90 remained in the lung, but were detected at very small amounts only in LALN; the latter is a finding, typically observed as a pathway for poorly soluble particles. The transport of particles from the alveolar lumen to the LALN implies a translocation across the alveolar epithelium to the interstitial space. The lymph system can take up the particles (in free form or mostly engulfed by macrophages) from the interstitium and subsequently the particles are filtered in the LALN. The blood compartment is not involved in this process. This transport mechanism differs from the translocation of particles to organs including a blood compartment passage.

**Fig. 3:**
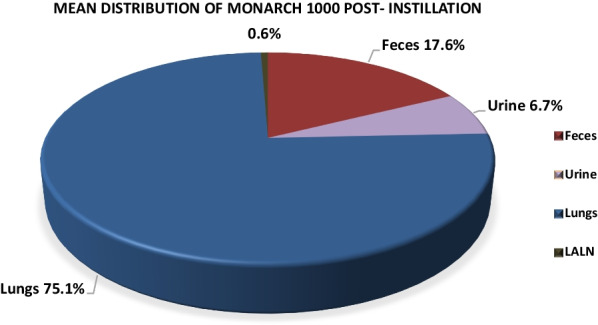
Toxicokinetics of ^7^Be-Monarch.® 1000 after intratracheal instillation. Feces/urine: day 1–3; Lungs/LALN: day 20 following administration. (% refer to the entire radioactivity detected in the rat body: = 100%)

**Fig. 4:**
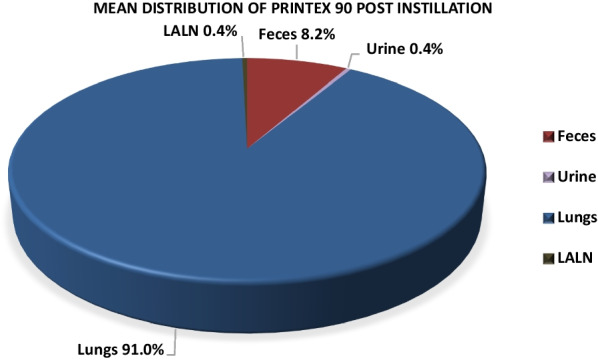
Toxicokinetics of ^7^Be-Printex.® 90 after intratracheal instillation. Feces/urine: day 1–3; Lungs/LALN: day 20 following administration. (% refer to the entire radioactivity detected in the rat body: = 100%)

In the lungs, our experiments show that the particles were primarily located in the alveolar macrophages. Centrifugation of BAL recovered in animals exposed to ^7^Be-Printex® 90, revealed that the isotope activity was entirely located in the cell compartment, indicating that it had been engulfed by alveolar macrophages. Overall, ^7^Be-Monarch® 1000 and ^7^Be-Printex® 90 were not systemically available after deposition in lungs (Figs. 3-4).

The toxicokinetic behaviour of carbon black following pulmonary exposure has been studied by other working groups. Niwa et al. [16] reported an inhalation study of carbon black in rats at a high aerosol concentrations (15 mg/m^3^ for 4 weeks). Evidence of translocation of inhaled carbon black was not found. The authors concluded that likely, inhaled carbon black particles remain in the lung and do not exert direct adverse effects on extrapulmonary tissues. However, others have reported the detection of particulate substances in systemic organs following intratracheal exposure to carbon black. Using brightfield microscopy, an optical imaging methodology, Modrzynska et al. [6, 17] reported the detection of carbon black particles in the liver of mice. Under the conditions of our study, we found no evidence that carbon black particles reach the liver. Concentrations of the ^7^Be-isotope in the liver were within the background noise of the measurement. The difference in outcome of the mouse study [17] in comparison to our study findings may be a corollary of the different lung loads selected in the two studies. Whereas we chose a dose below lung overload levels (0.3 mg per rat), in the mouse study [17] a dose of 0.162 mg per mouse was intratracheally instilled. In consideration of the rat to mice lung weight ratio of approximately 6, the corresponding dose in the rat would be approximately 1 mg/rat, a dose which evidently is a mass/volume overload. Mice have been shown to exhibit lung particle overload and lung clearance retardation as well whereas inflammatory and fibrotic reactions are weaker [18]. In mice, one could speculate that under conditions of marked overload, which is associated with physiological pertubations, the moiety of free carbon black particles may possibly be increased, and some translocation of particles to the blood compartment and liver might occur.

**Fig. 5:**
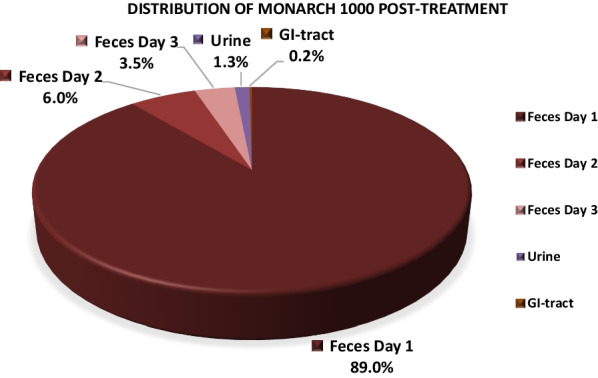
Toxicokinetics of ^7^Be-Monarch.® 1000 after oral gavage. Feces/urine: day 1–3; GI tract day 13 following administration. (% refer to the entire radioactivity detected in the rat body: = 100%)

**Fig. 6:**
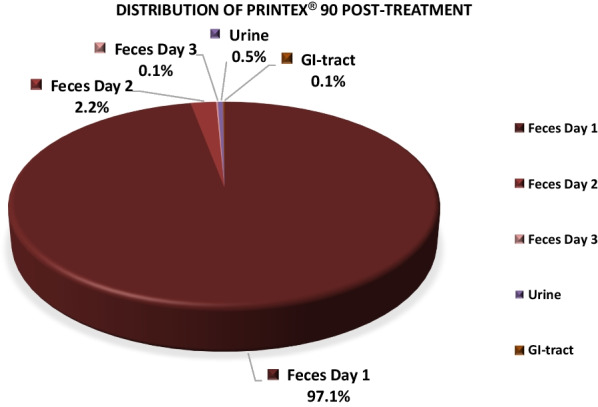
Toxicokinetics of ^7^Be-Printex.® 90 after oral gavage. Feces/urine: day 1–3; GI tract day 10 following administration (% refer to the entire radioactivity detected in the rat body: = 100%)

LeFevre et al. [7] studied the toxicokinetic behaviour in mice following oral (gavage) exposure to a single dose of 0.25 ml of ^7^Be-labelled carbon black suspension (28 mg carbon/ml). Similar to our findings, the authors also recorded no relevant translocation of carbon black into the systemic circulation in mice. The distribution of the isotope was determined in the animals 4 h and 1, 2, 5 and 14 days after exposure. Less than 0.014% of the applied radioactivity was found in non-intestinal tissues of the animals. In Peyer’s patches, less than 0.002% and 0.006% radioactivity were found in young and aged mice, respectively. According to the authors, the radioactivity in extra intestinal viscera and blood was negligibly low and practically all the label was excreted in the faeces [7]. The findings of these authors agree with the results we have seen in the rats orally exposed to labelled test items in this investigation. No significant evidence of translocation of carbon black particles across the GIT/blood barrier into the blood stream was detected. The majority of the labelled test materials was excreted in faeces within 3 days following gavage (Figs. 5-6). At necropsy, only 0.2% of measured activity remained in the GIT.

Borm & Driscoll [13] have discussed the need to “discriminate between clearance impairment caused by toxicity versus that caused by volumetric overload of macrophages. Intrinsic toxicity, such as occurs with crystalline quartz, may kill macrophages or impair their function, but this is an entirely different adverse outcome pathway (AOP) from the lung overload that Morrow described”. It has been observed that high doses of poorly soluble particles like carbon black, when administered into the lungs of small laboratory animals will cause lung overload leading to the disruption of the physiological integrity of the lungs. Therefore, when designing our study, we decided to simulate non-overload conditions to avoid any impairment of the normal physiological clearance capabilities in the lung of the rat. Under such conditions, the toxicokinetic data demonstrate that carbon black remains only in the target organs of administration (i.e. lungs and GI-tract) and does not become systemically available.

This study contributes to the debate of whether or not carbon black can translocate into systemic circulation following exposure and cause systemic effects, as has been reported by others. However, the findings in this study provide no support for either of these reported events.

## Conclusions

The results of both administration modes indicate that the two carbon black grades acted predominantly as microscaled agglomerated aggregates, not as individual nanoparticles. Under these experimental conditions, the two CB samples were not shown to translocate beyond the lung or the GI tract into the blood compartment. In the intratracheal instillation study besides lungs, ^7^Be-Printex® 90 and ^7^Be-Monarch® 1000 were detected at very small amounts only in LALN; this latter finding is consistent with what has been reported for other poorly soluble particles. Overall, under the radiolabel conditions and considering the analytical LOQ of the measurement method applied in this study, the two carbon black samples did not show an evident systemic availability after deposition in lungs or stomach.

## Supplementary Information

**Additional file 1.** Radioactivity measurements in organs/tissues of female and male rats.

## Data Availability

The datasets generated and analyzed during the current study are available from the corresponding author on reasonable request.

## Abbreviations

AAF: Artificial alveolar fluid
ADME: Absorption/Distribution/Metabolism/Elimination
ALF: Artificial lysosomal fluid
AOP: Adverse outcome pathway
Be: Beryllium
BAL(F): Bronchoalveolar lavage (fluid)
Cpm: Counts per minute
DLS: Dynamic light scattering
GI(T): Gastro-intestinal (tract)
hr(s): Hour(s)
IT: Intratracheal instillation
LALN: Lung-associated lymph nodes
LOQ: Limit of quantitation
N: Number
NP: Nanoparticles
OECD: Organization for economic co-operation and development
OG: Oral gavage
PSLT: Poorly soluble low toxicity
TG: Technical guideline
TK: Toxicokinetic
