# Supplementary material for: Toxicokinetic study following intratracheal instillation or oral gavage of two [7Be]-tagged carbon black samples
Source: Part Fibre Toxicol. 2022 Oct 14;19:63. doi: 10.1186/s12989-022-00504-8 (PMC9569049; doi:10.1186/s12989-022-00504-8)
Supplement: Supplementary file 1 — Additional file 1. Radioactivity measurements in organs/tissues of female and male rats. [file 12989_2022_504_MOESM1_ESM.docx]

Supplemental Table 1: Activity measurements in organs/tissues of female rats following intratracheal instillation of ^7^Be-Monarch^®^ 1000

| **Day 20**  **post-treatment**  **FEMALES** | Activity  (cpm)  measured | | Activity  (%)  Calculated | |
| --- | --- | --- | --- | --- |
| Animal-No.  **1201-1204** | Mean | SD | Mean | SD |
| Lungs | 2044.6 | 795.5 | 36.96 | 11.51 |
| Fat | 0.38 | 0.75 | 0 |  |
| Ovaries | 0.08 | 0.15 | 0 |  |
| Uterus | 0.70 | 0.82 | 0 |  |
| Spleen | 1.98 | 2.57 | 0 |  |
| Adrenals | 1.20 | 2.14 | 0 |  |
| Kidney | 0.28 | 0.43 | 0 |  |
| Stomach | 0.80 | 0.68 | 0 |  |
| Intestine | 0.08 | 0.15 | 0 |  |
| Colon | 0.05 | 0.10 | 0 |  |
| Thymus | 0.80 | 0.93 | 0 |  |
| Heart | 0.20 | 0.40 | 0 |  |
| LALN | 16.35 | 15.07 | 0.30 | 0.25 |
| Tongue | 1.95 | 2.39 | 0 |  |
| Eyes | 0.85 | 1.06 | 0 |  |
| Brain | 0.13 | 0.25 | 0 |  |
| Ears | 2.20 | 4.40 | 0 |  |
| Residual carcass | 0.30 | 0.60 | 0 |  |
| Muscle | 1.20 | 2.08 | 0 |  |
| Femur | 2.78 | 2.67 | 0 |  |
| Bone marrow | 0.63 | 1.25 | 0 |  |
| Liver I | 1.10 | 2.20 | 0 |  |
| Liver II | 0 | 0 | 0 |  |
| Total blood | 1.08 | 2.15 | 0 |  |

(% refer to the nominal dose:

Radioactivity of the 0.3 mg carbon black suspension in 300 µl saline used for administration = 100%)

Supplemental Table 2: Activity measurements in organs/tissues of female rats following intratracheal instillation of ^7^Be-Printex 90^®^

| **Day 20**  **post-treatment**  **FEMALES** | Activity  (cpm)  measured | | Activity  (%)  calculated | |
| --- | --- | --- | --- | --- |
| Animal-No.  **2201-2204** | Mean | SD | Mean | SD |
| Lungs | 19413 | 7343 | 63.4 | 24.3 |
| Fat | 1.1 | 1.7 | 0 |  |
| Ovaries | 0.9 | 0.4 | 0 |  |
| Uterus | 0.9 | 0.6 | 0 |  |
| Spleen | 0.1 | 0.3 | 0 |  |
| Adrenals | 1.2 | 1.8 | 0 |  |
| Kidney | 8.0 | 11.8 | 0 |  |
| Stomach | 1.8 | 1.6 | 0 |  |
| Intestine | 1.8 | 1.2 | 0 |  |
| Colon | 1.0 | 1.2 | 0 |  |
| Thymus | 1.8 | 1.9 | 0 |  |
| Heart | 1.9 | 1.8 | 0 |  |
| LALN | 112.6 | 107.9 | 0.3 | 0.2 |
| Tongue | 2.4 | 3.0 | 0 |  |
| Eyes | 0.6 | 1.2 | 0 |  |
| Brain | 2.4 | 2.4 | 0 |  |
| Ears | 2.5 | 1.8 | 0 |  |
| Residual carcass | 3.0 | 2.1 | 0 |  |
| Muscle | 1.5 | 1.9 | 0 |  |
| Femur | 0.8 | 0.5 | 0 |  |
| Bone marrow | 1.7 | 2.3 | 0 |  |
| Liver I | 3.2 | 2.7 | 0 |  |
| Liver II | 0.1 | 0.1 | 0 |  |
| Total blood | 1.5 | 1.6 | 0 |  |

(% refer to the nominal dose:

Radioactivity of the 0.3 mg carbon black suspension in 300 µl saline used for administration = 100%)

Supplemental Table 3: Activity measurements in organs/tissues of male rats following intratracheal instillation of ^7^Be-Monarch^®^ 1000

| Day 20  post-treatment  **MALES** | Activity  (cpm)  measured | | Activity  (%)  Calculated | |
| --- | --- | --- | --- | --- |
| Animal-No.  1101-1104 (n=4) | Mean | SD | Mean | SD |
| Lungs | 2569 | 60 | 46.20 | 11.00 |
| Testes | 2.18 | 2.80 | 0 |  |
| Epididymides | 2.53 | 1.20 | 0 |  |
| Kidney | 2.08 | 1.65 | 0 |  |
| Liver I | 1.15 | 1.60 | 0 |  |
| Liver II | 0.95 | 1.90 | 0 |  |
| Total blood | 1.93 | 1.59 | 0 |  |

(% refer to the nominal dose:

Radioactivity of the 0.3 mg carbon black suspension in 300 µl saline used for administration = 100%)

Supplemental Table 4: Activity measurements in organs/tissues of male rats following intratracheal instillation of ^7^Be-Printex 90^®^

| Day 20  post-treatment  **MALES** | Activity  (cpm)  Measured | | Activity  (%)  Calculated | |
| --- | --- | --- | --- | --- |
| Animal-No.  2101-2104 (n=4**)** | Mean | SD | Mean | SD |
| Lungs | 7839 | 1207 | 41.33 | 7.74 |
| Testes | 0.80 | 0.61 | 0 |  |
| Epididymides | 0.70 | 0.82 | 0 |  |
| Kidney | 1.40 | 1.28 | 0 |  |
| Liver I | 3.50 | 2.07 | 0 |  |
| Liver II | 1.58 | 1.73 | 0 |  |
| Total blood | 0.15 | 0.30 | 0 |  |

(% refer to the nominal dose:

Radioactivity of the 0.3 mg carbon black suspension in 300 µl saline used for administration = 100%)

Supplemental Table 5: Activity measurements in organs/tissues of female rats following gavage administration of ^7^Be-Monarch^®^ 1000

| **Day 13**  **post-treatment**  **FEMALES** | Activity  (cpm)  measured | | Activity  (%)  calculated | |
| --- | --- | --- | --- | --- |
| Animal-No.  **4201-4204** | Mean | SD | Mean | SD |
| Lungs | 3.1 | 3.7 | 0 | not given |
| LALN | 3.1 | 0.9 | 0 |  |
| Fat | 3.4 | 1.7 | 0.05 |  |
| Ovaries | 3.6 | 2.9 | 0.05 |  |
| Uterus | 2.5 | 1.8 | 0 |  |
| Spleen | 2.4 | 2.0 | 0 |  |
| Adrenals | 0.2 | 0.5 | 0 |  |
| Kidney | 4.0 | 3.4 | 0.05 |  |
| Stomach A | 5.5 | 5.4 | 0.05 |  |
| Intestine B | 3.9 | 2.2 | 0.05 |  |
| Colon C | 4.0 | 3.6 | 0 |  |
| A + B + C | 13.7 | 8.7 | 0.2 |  |
| Thymus | 1.8 | 1.3 | 0 |  |
| Heart | 6.6 | 5.0 | 0.08 |  |
| Tongue | 1.9 | 2.2 | 0 |  |
| Eyes | 0.8 | 1.7 | 0 |  |
| Brain | 3.3 | 2.2 | 0 |  |
| Ears | 1.0 | 1.3 | 0 |  |
| Skin | 4.0 | 3.1 | 0.05 |  |
| Muscle | 3.8 | 3.4 | 0.05 |  |
| Femur | 2.2 | 4.1 | 0 |  |
| Bone marrow | 1.9 | 1.9 | 0 |  |
| Liver | 4.7 | 2.0 | 0.05 |  |
| Total blood | 0.9 | 0.9 | 0 |  |

(% refer to the nominal dose:

Radioactivity of the 0.3 mg carbon black suspension in 1.5 ml water used for administration = 100%)

Supplemental Table 6: Activity measurements in organs/tissues of female rats following gavage administration of ^7^Be-Printex 90^®^

| **Day 10**  **post-treatment**  **FEMALES** | Activity  (cpm)  measured | | Activity  (%)  calculated | |
| --- | --- | --- | --- | --- |
| Animal-No.  **3201-3204** | Mean | SD | Mean | SD |
| Lungs | 1.0 | 0.7 | 0.0 | not given |
| LALN | 0.7 | 1.2 | 0.0 |  |
| Fat | 0.2 | 0.3 | 0.0 |  |
| Ovaries | 0.4 | 0.7 | 0.0 |  |
| Uterus | 0.0 | 0.0 | 0.0 |  |
| Spleen | 1.5 | 1.3 | 0.0 |  |
| Adrenals | 0.4 | 0.7 | 0.0 |  |
| Kidney | 0.1 | 0.1 | 0.0 |  |
| Stomach A | 1.0 | 0.4 | 0.0 |  |
| Intestine B | 1.4 | 0.8 | 0.0 |  |
| Colon C | 1.3 | 1.0 | 0.0 |  |
| A + B + C | 3.7 | 0.9 | 0.0 |  |
| Thymus | 2.0 | 2.1 | 0.0 |  |
| Heart | 0.1 | 0.2 | 0.0 |  |
| Tongue | 0.0 | 0.0 | 0.0 |  |
| Eyes | 0.6 | 0.8 | 0.0 |  |
| Brain | 0.2 | 0.3 | 0.0 |  |
| Ears | 1.1 | 1.9 | 0.0 |  |
| Skin | 1.1 | 1.4 | 0.0 |  |
| Muscle | 0.3 | 0.6 | 0.0 |  |
| Femur | 0.7 | 0.8 | 0.0 |  |
| Bone marrow | 0.4 | 0.8 | 0.0 |  |
| Liver | 0.7 | 0.6 | 0.0 |  |
| Total blood | 0.7 | 1.2 | 0.0 |  |

(% refer to the nominal dose:

Radioactivity of the 0.3 mg carbon black suspension in 1.5 ml water used for administration = 100%)

Supplemental Table 7: Activity measurements in organs/tissues of male rats following

gavage administration of ^7^Be-Monarch^®^ 1000

| Day 20  post-treatment  **MALES** | Activity  (cpm)  Measured | | Activity  (%)  Calculated |
| --- | --- | --- | --- |
| Animal-No.  4101-4104 | Mean | SD | Mean |
| Testes | 0.03 | 0.05 | 0.0 |
| Epididymides | 0.80 | 0.98 | 0.0 |
| Kidney | 1.10 | 1.51 | 0.0 |
| Liver I | 2.28 | 2.03 | 0.0 |
| Total blood | 3.25 | 2.39 | 0.0 |
| Stomach | 4.60 | 2.90 | 0.1 |
| Intestine | 2.33 | 1.20 | 0.0 |

(% refer to the nominal dose:

Radioactivity of the 0.3 mg carbon black suspension in 1.5 ml water used for administration = 100%)

Supplemental Table 8: Activity measurements in organs/tissues of male rats following gavage administration of ^7^Be- Printex 90^®^

| Day 20  post-treatment  **MALES** | Activity  (cpm)  Measured | | Activity  (%)  Calculated |
| --- | --- | --- | --- |
| Animal-No.  3101-3104 | Mean | SD | Mean |
| Testes | 1.45 | 2.03 | 0 |
| Epididymides | 0.70 | 0.84 | 0 |
| Kidney | 0.58 | 1.15 | 0 |
| Liver I | 0.48 | 0.66 | 0 |
| Total blood | 0.20 | 0.40 | 0 |
| Stomach | 0.00 | 0.00 | 0.1 |
| Intestine | 0.33 | 0.39 | 0 |

(% refer to the nominal dose:

Radioactivity of the 0.3 mg carbon black suspension in 1.5 ml water used for administration = 100%)
